# Prevalence of Low Self-esteem and Mental Distress among Undergraduate Medical Students in Jimma University: A Cross-Sectional Study

**DOI:** 10.4314/ejhs.v31i3.14

**Published:** 2021-05

**Authors:** Netsanet Workneh Gidi, Ararsa Horesa, Habtemu Jarso, Workineh Tesfaye, Gudina Terefe Tucho, Mubarek Abera, Jemal Abafita

**Affiliations:** 1 Department of Pediatrics and Child Health, Jimma University, Jimma, Ethiopia; 2 Department of Epidemiology, Jimma University, Jimma, Ethiopia; 3 Department of Environmental health sciences and technology, Jimma University, Jimma, Ethiopia; 4 Department of Psychiatry, Jimma University, Jimma, Ethiopia; 5 Department of Economics, Jimma University, Jimma, Ethiopia

**Keywords:** Mental distress, low self-esteem, medical education

## Abstract

**Background:**

Medical students often experience chronic stress. Self-esteem is one of the most important factors in the process of psychosocial growth and has remarkable effect on thoughts, feelings, values, and goals. The aim of this study was to assess the prevalence and associated factors of low self-esteem (LSE) and mental distress among Medical Students of Jimma University.

**Method:**

This cross-sectional study was conducted in Jimma University from June to July, 2018. Self-esteem was measured with Rosenberg self-esteem scale. Self-administered Short form with General Health Questionnaire was used to assess presence of mental distress.

**Result:**

Out of 422 students enrolled to the study, 279 (66.1%) were male, and 413 (97.9 %) were 18 to 25 years of age. The prevalence of LSE and mental distress were 19.0%, and 19.7 %, respectively. Students who had LSE had 5 times higher risk of having mental distress, AOR= 5.1 (95% CI, 2.9–8.9). Moreover, female students had higher risk of developing mental distress (AOR=1.9, 95% CI, 1.1–3.3). Students who had poor social support were 4.3 times at higher risk of developing LSE, AOR=4.3 (95% CI, 1.9–9.8). Those who reported to have poor academic performance were also more likely to have LSE AOR= 3.7 (95% CI, 1.3–10.0).

**Conclusion:**

One in five medical students had LSE and it is strongly associated with metal distress. Female students were at higher risk of mental distress. Preventive, curative and rehabilitative mental health services should be available for medical students with particular attention to those with poor social support.

## Introduction

Self-esteem is defined as the person's overall subjective view of one's own worth, which is related to a feeling of personal aptitude, success and pride, or to a feeling of despair and shame (1, 2). Self-esteem is considered very important for mental health; which influences the emotional states, general adaptability to life-challenges and resilience to stress during lifetime (3, 4). Undergraduate students are more prone to mental illness as they are going through physiologic and social changes that happen in late adolescence and early adulthood (5).

Positive psychological resources like, high self-esteem influences person's initiative, motivation and success in academic and professional activities (1, 6, 7). Individuals who have lived in disadvantaged situation, otherwise have high self-esteem, are motivated to fight for a better life (8). Adolescents and young adults may show low self-esteem (LSE) because of a culture that promotes children to be silent and obedient, where non-assertiveness is considered a sign of decency (9, 10). Education, including life skill training (LST) improves self-esteem (11, 12). Several organizations involved in promotion of life skills development in young people in Ethiopia often focus on problems such as abuse, HIV/AIDS, poverty; usually target the urban youth and lack sustainability (13).

Medical students often experience chronic stress due to excessive workload, difficulties with time management for studying, conflicts in work–life balance and relationships; that could affect academic achievement as well as their professional life (14–16). High prevalence of anxiety, depression and stress has been reported by several authors, and it is in the range of 3% to 63% (14, 17–22).

Students with mental illness are likely to experience poor academic performance. Gender differences in prevalence of mental distress have been depicted in literatures. Female students suffer from depression and anxiety more than their male colleagues (14, 20). Even though there were prior studies on medical students stress and its association with substance use, and common mental illnesses in undergraduate medical students in Jimma University, the prevalence of low self-esteem and its association with mental distress is unknown (23, 24). The aim of this study was to assess the prevalence and associated factors of low self-esteem and mental distress among Medical Students of Jimma University.

## Methods

**Study setting and design**: This cross-sectional study was conducted from June 2018 to July 2018 in Jimma University. Jimma Medical Center is one of the tertiary hospitals, serving about 15 million population in southwest of Ethiopia. The medical school was founded in 1983, in addition to the undergraduate training; there are residency programs in all the major departments.

**Study population**: Undergraduate Medical students attending Jimma University were the study population.

**Sampling and Data collection**: The total number of medical students in Jimma University at the time of the study was 1627. Sample size was calculated using a single population proportion formula with following assumptions: Prevalence (p=0.5), margin of sampling error to be tolerated= 5%, and 95% confidence interval of certainty. The sample size for this study was 384. Then by adding 10% non-response rate the final sample size was 422. Stratified sampling procedure was done to decide the number of students needed from each year of training. Data was collected in a class room setting using a self-report questionnaire prepared for the study.

**Instruments and measurements**: The questionnaire included Socio-demographic data, Rosenberg self-esteem scale (RSE), a short General Health Questionnaire (GHQ-12) and Oslo social support scale-3. RSE is a commonly used tool which has been found to be a reliable instrument for measuring self-esteem across different cultures (25). It consists of 10 items, for positively worded items (items 1, 2, 4, 7, 9, 10) 4-point Likert type scale (0 – strongly disagree to 3 – strongly agree) were used; and for the negatively worded items (3, 5, 6, and 8) the scoring was reversed. A total score of 22 is considered the average while high self-esteem is 25 to 30. Normal range is 15 to 25, and a score less than 15 is generally considered LSE (Morris Rosenberg) (26).

Social support was assessed using Oslo Social Support Scale 3 (OSSS-3). OSSS-3 is widely used for epidemiological studies. The tool consists of three questions; “how many people are so close to you that you can count on them if you have great personal problems?”, “How much interest and concern do people show in what you do?”, and “How easy is it to get practical help from family or relatives if you should need it?”. The sum score ranges from 3 to 14, 3–8 indicates poor social support; 9–11 moderate social support and 12–14 strong social support (27).

Psychometric Properties of GHQ, as a Screening tool for anxiety and depressive symptoms was studied for Ethiopian young adults and showed good internal consistency (Cronbach's alpha: 0.83) (28). The GHQ-12 assesses how respondents felt during the last four weeks, those problems related with sleep and appetite, subjective experiences of stress, tension, or sadness. Mastering of daily problems, decision making and self-esteem are also included. Response choices included are: less than usual, not more than usual, more than usual and much more than usual. A score of 0 is given for the first two choices and 1 for the next two. The maximum possible score is 12 with higher scores suggesting higher mental distress. A score ≥5 on the GHQ-12 scale are suggestive of common psychiatric disorders (29)

**Statistical analyses**: The statistical analysis was done by SPSS statistical software version 23. The total scores of the above tools were added up, categorized according to the recommended cut offs. Following descriptive analysis, the chisquare test was used to check the cell count adequacy before performing binary logistic regression. Factors that could be associated with the dependent variables were identified from bivariate binary logistic regression (P-value < 0.2). Multivariable binary logistic regression was performed to identify factors independently associated with outcome variables (P-value < 0.05).

**Ethical consideration**: Data collection was conducted after ethical approval was obtained from Ethical Review Board (IRB) of Institute of Health, Jimma University. Information sheet was given together with the questionnaire to the participants, the names of the participants were not asked. Participants were informed that they have a right to decline participating in the study and filling the questionnaire indicates informed consent.

## Results

Out of 422 students enrolled to the study 279 (66.1%) were male, and 413 (97.9 %) were 18 to 25 years of age. Majority, 328 (77.7%) of them were living in urban area before joining medical school. Half of the students, 213 (50.5%) are from Oromo ethnic group and only 11 (2.6%) of them were married. One fourth of the students joined medical school pressurized by parents and relatives. Majority of the students reported getting adequate financial support, while 69 (16.4%) of them rated their financial support as barely adequate or inadequate (Table 1). Only 65 (15.4%) of the students reported ever using substance/s, out of which use of alcohol was the commonest 34 (8.1%). Less than half, 170 (40.3%) of the students had strong social support, and one in 10 had poor social support.

**Table 1:** Characteristics of study participants, undergraduate medical students in Jimma University

Variables		Frequency(N=422)	Percentage
Age	18–20	111	26.3
	21–25	302	71.6
	>25	9	2.1
Gender	Male	279	66.1
	Female	143	33.9
Residence prior to joining	Urban	328	77.7
university	Rural	94	22.3
Ethnicity	Oromo	213	50.5
	Amhara	150	35.5
	Somali	13	3.1
	Tigre	7	1.7
	Gurage	22	5.2
	Other	17	4.0
Marital Status	Married	11	2.6
	Single	411	97.4
Year of study	Year one	48	11.4
	Year two	63	14.9
	Year three	96	22.7
	Year four	70	16.6
	Year five	84	19.9
	Year six	61	14.5
Working part time	Yes	17	4.0
	No	405	96.0
Rating of financial support	Highly Adequate	68	16.1
	Adequate	277	65.6
	Barely adequate	37	8.8
	Inadequate	32	7.6
Maternal schooling	No school	77	18.2
	Some primary school	72	17.1
	Completed primary school	32	7.6
	Secondary school	86	20.4
	Higher education	155	36.7
Father's schooling	Never been to school	47	11.1
	Completed primary school	91	21.6
	Secondary school	57	13.5
	Higher education	227	53.8
Reason for joining medicine	Self interest	314	74.4
	Pressurized by family and relatives	108	25.6

The study participants mean (SD) of RSE was 19.5 (4.8), the majority of the students had normal or high self-esteem while the prevalence of LSE was 19.0%. (Table 2). Those who reported poor academic performance were also more likely to have LSE, AOR = 3.7, (95% CI: 1.3–10.0). The use of social media was positively associated with self-esteem, but on multivariable analysis the association was not significant (Table 3). Other socio-demographic factors such as age, gender, place of residence, financial support they have, parental educational status were not associated with LSE. Poor social support which was assessed by Oslo social support scale and subjective academic performance of the students were independently associated with LSE. Students who had poor social support were 4.3 times at higher risk of developing LSE, AOR = 4.3, (95% CI:1.9–9.8).

**Table 2:** Level of self-esteem, social support and Prevalence of mental distress among undergraduate medical students in Jimma University

Variables		Number	Percent
**Self-esteem**	low	80	19.0
	normal	294	69.7
	high	48	11.4
**Social support**	poor	42	10.0
	moderate	210	49.8
	strong	170	40.3
**Mental distress**	Present	83	19.7
	Absent	339	80.3

**Table 3:** Multivariable logistic regression analysis of factors associated with low self-esteem among undergraduate medical students in Jimma University

Variables		Low self-esteem		COR (95% C.I)	AOR (95% C.I)
		Yes	No		
Year of education (continuous variable)				1.12 (0.93, 1.31)	1.1 (1.0, 1.3)
Social support	Strong	18	152	1	1
	Poor	**16**	**26**	**5.2 (2.35, 11.5)**	**4.3 (1.9,9.8)**
	Moderate	46	164	2.1 (1.08,4.4)	1.8 (0.9, 3.7)
Subjective academic performance	Very good	10	60	1	1
	Poor	**16**	**25**	**3.8 (1.5, 9.6)**	**3.7(1.3, 10.0)**
	Good	**54**	**257**	**3.0 (1.5, 6.1)**	**2.8 (1.3,5.9)**
Reason for joining medicine	Self interest	54	260	1	1
	Pressurized by family/relatives	26	82	1.5(0.8,2.5)	1.2 (0.7,2.3)
Use of social media	Yes	65	311	1	1
	No	15	31	2.3 (1.2,4.5)	1.9 (1.0, 4.0)

The prevalence of mental distress assessed by a short form of general health questionnaire was 19.7 %. On multivariate logistic regression analysis, gender and LSE were independently associated with higher risk of mental distress. Female students were about twice at risk of developing mental distress than their male colleagues, AOR = 1.9, (95% CI: 1.1–3.3). Students who had LSE had 5 times higher risk of having mental distress, AOR = 5.1, (95% CI: 2.9–8.9). Poor social support was found to be associated with higher risk of mental distress, however when OR was adjusted for other variables, there was no statistically significant association (Table 4).

**Table 4:** Multivariable logistic regression analysis, factors associated with mental distress among undergraduate medical students in Jimma University

Variables		Mental distress		COR (95% C.I)	AOR (95% C.I)
				
		Yes	No		
Gender	Male	47	232	1	1
	Female	36	107	1.7 (1.0, 2.7)	1.9 (1.1, 3.3)
residence	urban	61	267	1	1
	rural	22	72	1.3 (0.8, 2.3)	1.5 (0.8, 2.7)
Substance use	No	67	290	1	1
	Yes	16	49	0.7 (0.4,1.3)	0.7 (0.3, 1.4)
Social support	Strong	24	146	1	1
	poor	14	28	3.0 (1.4, 6.6)	1.9 (0.8, 4.4)
	moderate	45	165	1.6 (1.0, 2.8)	1.5 (0.8, 2.7)
Reason for joining medicine	Self interest	56	258	1	1
	Pressurized by family/relatives	27	81	1.5 (0.9, 2.5)	1.2 (0.7, 2.2)
Self-esteem	Normal or high	45	297	1	1
	Low	38	42	6.0 (3.4, 10.2)	5.1 (2.9, 8.9)
Subjective academic performance	Very good	10	60	1	1
	good	57	254	1.3 (0.6, 2.8)	0.4(0.1, 1.2)
	poor	16	25	3.8 (1.5, 9.6)	0.5(0.2, 1.1)

## Discussion

Medical education and training is often perceived very stressful, and may directly contribute for psychological distress (15, 16, 30). Similarly, Melaku et al. reported 52.4% prevalence of stress among undergraduate medical students in Jimma University (23). In the current cross sectional study the prevalence of LSE was 19.0%, and 19.7% of the students scored ≥ 5 on GHQ-12, which suggests mental distress, presence of one or more common psychiatric disorders (29).

Poor social support and unsatisfactory subjective academic performance were associated with LSE. This finding is in accordance with a meta-analytic review by Twenge et al., they found that individuals with higher socioeconomic status report higher selfesteem (31). Students with LSE could have lesser desire to be perceived as competent in their trainings, in the contrary those who have high self-esteem may keep the positive attitude and show resilience following failure (32). Psychosocial stability is one of the factors that determine the level of preparedness for examinations and scoring satisfactory grade (33). The 19.0% prevalence of LSE is considerably high, as medical students are generally considered high achievers with at least average or high intellectual capacity (34). LSE is strongly associated with mental distress. Students who had LSE had a fivefold risk of having mental distress. A similar prevalence of LSE (23.4%) and inverse relationship with depression, anxiety and stress was observed in dental and medical students in Jeddah Saudi Arabia (35).

The prevalence of mental distress in our study is similar to the prevalence of mental disorder estimated in general population among young adults in their twenties (19% to 32%) (30, 34, 35). A study conducted in Jimma University in 2015 by Kerebih et al. (24), had also revealed 35.2% prevalence of common mental disorders among medical students (2). Sreeramareddy et al. found a similar 21% prevalence of psychological morbidity in Nepalese medical students (22), and a slightly lower prevalence of psychiatric disorders was reported by Tysson et al from Norway (17.2%) (21) and Hardeman et al. from USA (3% to 17%) among first year medical students (20). Higher prevalence of mental distress has been reported by several authors from certain countries; 63% was reported by Abdulghani et al from Saudi Arabia (17); 49.5%, by Jafari et al from Iran (14); 48%, Aktekin et al Turkey (19); 29.6%, Almeida et al from Brazil (18); this could be due to the different tools used to diagnose mental distress or related to the diverse teaching culture of medical schools.

Most of the socio-demographic variables studied were not associated with mental distress, while gender and LSE were strongly associated with mental distress independently. Being female student was associated with two fold higher risk of having mental distress compared to male students. This finding is in line with previous literatures Jafari et al. (14), Hardman et al. (20), Rosal et al. (16) and Amr et al. (36); but in contrast to our finding Aboalshamat et al (35) and Tysson et al. (21) found no gender difference in prevalence of mental distress among medical students. The reason why female students are prone to mental distress needs further investigation.

The prevalence of LSE among medical students in Jimma University is considerably high and it is strongly associated with metal distress. Female students are at higher risk of mental distress. Students who have poor social support and perceive their academic performance as unsatisfactory were more likely to have LSE. Screening students for LSE and mental distress and timely intervention through psychological counseling programs is needed, that might contribute for better academic performance and improved wellbeing in subsequent years of their lives. Raising awareness on mental health problems and availing preventive, curative and rehabilitative mental health services for medical students is highly needed to prevent mental illness in future doctors.

## References

[1] Baumeister RF, Campbell JD, Krueger JI, Vohs KD (2003). Does high self-esteem cause better performance, interpersonal success, happiness, or healthier lifestyles?. Psychol Sci.

[2] Rosenberg MJA, package ctM (1965). Rosenberg selfesteem scale (RSE).

[3] Baumeister RF, Vohs KD (2003). Willpower, choice, and self-control. Time and decision: Economic and psychological perspectives on intertemporal choice.

[4] Dishman RK, Hales DP, Pfeiffer KA, Felton GA, Saunders R, Ward DS (2006). Physical selfconcept and self-esteem mediate cross-sectional relations of physical activity and sport participation with depression symptoms among adolescent girls. Health Psychol.

[5] Radeef AS, Faisal GG (2019). Low Self-esteem and its Relation with Psychological Distress among Dental Students.

[6] Stupnisky RH, Renaud RD, Perry RP, Ruthig JC, Haynes TL, Clifton RA (2007). Comparing selfesteem and perceived control as predictors of first-year college students' academic achievement. Soc Psychol Educ.

[7] Ciarrochi J, Heaven PC, Davies F (2007). The impact of hope, self-esteem, and attributional style on adolescents' school grades and emotional wellbeing: A longitudinal study. J Res Pers.

[8] Wang Y, Zhang L, Kong X, Hong Y, Cheon B, Liu J (2016). Pathway to neural resilience: Self-esteem buffers against deleterious effects of poverty on the hippocampus. Hum. Brain Mapp.

[9] Rudy D, Grusec JE (2006). Authoritarian parenting in individualist and collectivist groups: Associations with maternal emotion and cognition and children's self-esteem. J Fam Psychol.

[10] Guarcello L, Rosati F (2007). Child labor and youth employment: Ethiopia Country Study. World Bank Social Protection Working Paper Series.

[11] Maryam E, Davoud MM, Zahra G (2011). Effectiveness of life skills training on increasing self-esteem of high school students. Procedia Soc Behav Sci.

[12] Yadav P, Iqbal N (2009). Impact of life skill training on self-esteem, adjustment and empathy among adolescents. J Indian Acad Appl Psychol.

[13] Kibret BT (2016). Life Skills Training (LST) Program for Young People: Justifications, Foundations and Contents. Int J Sch Cog Psychol.

[14] Jafari N, Loghmani A, Montazeri A (2012). Mental health of medical students in different levels of training. Int J Prev Med.

[15] Saipanish R (2003). Stress among medical students in a Thai medical school. Med Teach.

[16] Rosal MC, Ockene IS, Ockene JK, Barrett SV, Ma Y, Hebert JR (1997). A longitudinal study of students' depression at one medical school. Acad Med.

[17] Abdulghani HM, AlKanhal AA, Mahmoud ES, Ponnamperuma GG, Alfaris EA (2011). Stress and its effects on medical students: a cross-sectional study at a college of medicine in Saudi Arabia. J Health Popul Nutr.

[18] Almeida AD, Godinho TM, Bitencourt AG, Teles MS, Silva AS, Fonseca DC, Barbosa DB, Oliveira PS, Costa-Matos E, Soares AM, Abade B (2007). Common mental disorders among medical students. Braz J Psychiatry.

[19] Aktekin M, Karaman T, Senol YY, Erdem S, Erengin H, Akaydin M (2001). Anxiety, depression and stressful life events among medical students: a prospective study in Antalya, Turkey. Med Educ.

[20] Hardeman RR, Przedworski JM, Burke SE, Burgess DJ, Phelan SM, Dovidio JF (2015). Mental well-being in first year medical students: A comparison by race and gender. J Racial Ethn Health Disparities.

[21] Tyssen R, Vaglum P, Grønvold NT, Ekeberg Ø (2001). Factors in medical school that predict postgraduate mental health problems in need of treatment. A nationwide and longitudinal study. Med Educ.

[22] Sreeramareddy CT, Shankar PR, Binu VS, Mukhopadhyay C, Ray B, Menezes RG (2007). Psychological morbidity, sources of stress and coping strategies among undergraduate medical students of Nepal. BMC Med Educ.

[23] Melaku L, Mossie A, Negash A (2015). Stress among medical students and its association with substance use and academic performance. J Biomed Educ.

[24] Kerebih H, Ajaeb M, Hailesilassie H (2017). Common mental disorders among medical students in Jimma University, Southwest Ethiopia. African health sciences.

[25] García JA, y Olmos FC, Matheu ML, Carreño TP (2019). Self esteem levels vs global scores on the Rosenberg self-esteem scale. Heliyon.

[26] Nguyen DT, Wright EP, Dedding C, Pham TT, Bunders J (2019). Low self-esteem and its association with anxiety, depression, and suicidal ideation in Vietnamese secondary school students: a cross-sectional study. Front Psychiatry.

[27] Kocalevent RD, Berg L, Beutel ME, Hinz A, Zenger M, Härter M (2018). Social support in the general population: standardization of the Oslo social support scale (OSSS-3). BMC Psychol.

[28] Gelaye B, Tadesse MG, Lohsoonthorn V, Lertmeharit S, Pensuksan WC, Sanchez SE (2015). Psychometric properties and factor structure of the General Health Questionnaire as a screening tool for anxiety and depressive symptoms in a multi-national study of young adults. J Affect Disord.

[29] Araya R, Wynn R, Lewis G (1992). Comparison of two self administered psychiatric questionnaires (GHQ-12 and SRQ-20) in primary care in Chile. Soc Psychiatry Psychiatr Epidemiol.

[30] Haglund ME, aan het Rot M, Cooper NS, Nestadt PS, Muller D, Southwick SM (2009). Resilience in the third year of medical school: a prospective study of the associations between stressful events occurring during clinical rotations and student well-being. Acad Med.

[31] Twenge JM, Campbell WK (2002). Self-esteem and socioeconomic status: A meta-analytic review. Personality and social psychology review.

[32] Park LE, Crocker J, Kiefer AK (2007). Contingencies of self-worth, academic failure, and goal pursuit. Pers Soc Psychol Bull.

[33] Augustine Egwu O, Dimkpa U, Ogbonnaya Orji J, Ogbannaya Njoku C, Ogbonnia Eni E, Besong E (2011). Medical students - self-assessed confidence level before a major physiology examination: affective factors in a nigerian medical school. Acta Inform Med.

[34] Yesikar V, Guleri S, Dixit S, Rokade R, Parmar S (2015). Intelligence quotient analysis and its association with academic performance of medical students. Int J Community Med Public Health.

[35] Aboalshamat K, Jawhari A, Alotibi S, Alzahrani K, Al-Mohimeed H, Alzahrani M (2017). Relationship of self-esteem with depression, anxiety, and stress among dental and medical students in Jeddah, Saudi Arabia. Int J Med Dent.

[36] Amr M, El Gilany AH, El-Hawary A (2008). Does gender predict medical students' stress in Mansoura, Egypt?. Med Educ Online.

